# Two years outcomes of treating full-thickness macula hole associated with idiopathic macular telangiectasia type 2 by internal limiting membrane inverted flap technique: Case reports

**DOI:** 10.1097/MD.0000000000027078

**Published:** 2021-09-10

**Authors:** Shungo Nishiyama, Takeshi Iwase

**Affiliations:** Department of Ophthalmology, Akita University Graduate School of Medicine, Akita, Japan.

**Keywords:** fluorescein angiography, full-thickness macular hole, idiopathic macular telangiectasia type 2, internal limiting membrane with inverted flap, optical coherence tomography, pars plana vitrectomy

## Abstract

**Rational:**

Macular telangiectasia (MacTel) is an uncommon ocular disorder that can lead to legal blindness. MacTel type 2 is characterized by a bilateral loss of macular transparency, the presence of white crystals on the retina, aberrant blood vessel growth, and neurodegeneration of the macula. Full-thickness macular holes (FTMHs) are a prominent cause of vision reduction in MacTel type 2, and the standard care for an FTMH is pars plana vitrectomy (PPV) to restore the FTMH and best-corrected visual acuity (BCVA). However, surgical outcomes in previous reports were not good, with a lack of closure or a reopening of the FTMH, compared with those with an idiopathic FTMH. Thus, this study aimed to determine the surgical outcomes of PPV with the inverted ILM flap technique for the treatment of FTMHs with a 2-year postoperative follow-up in three patients with MacTel type 2.

**Patient concerns:**

This study involved 3 patients who had been diagnosed with MacTel type 2 at a local eye clinic and who was subsequently referred to our department for a more detailed examination.

**Diagnoses:**

Three patients were diagnosed with MacTel type 2 using dilated ophthalmoscopy, fluorescein angiography, and optical coherence tomography (OCT) in both eyes. A FTMH was developed and visual acuity decreased during follow-up period in all of the patients.

**Interventions:**

Each patient underwent PPV in 1 eye using the inverted ILM-flap technique, gas tamponade, and prone positioning.

**Outcomes:**

The FTMH was successfully closed in the 3 cases after the surgery. OCT showed that the FTMH remained closed at the last follow-up examination in 2 patients and vision improved to 20/20 and 20/25. In the other patient, the hole was closed temporarily after surgery, but was reopened at 6 months. The vision had improved to 20/60 until the hole was reopened, and it was 20/100 at the final follow-up examination.

**Lessons:**

Although only 3 patients were examined, the inverted ILM-flap technique may be an effective and safe method to close an FTMH in patients with MacTel type 2. However, the surgery cannot prevent the reopening of the hole when the retinal atrophy progresses.

## 1 Introduction

Macular telangiectasia (MacTel) is an uncommon ocular disorder that can lead to legal blindness. MacTel type 2 is characterized by a bilateral loss of macular transparency, the presence of white crystals on the retina, aberrant blood vessel growth, and neurodegeneration of the macula.^[1–4]^ In more advanced cases, subretinal neovascularization can develop, which leads to a more severe reduction of vision.^[1]^ The cause of MacTel type 2 is still unknown, and no treatment exists to prevent the progressive loss of central vision.

Full-thickness macular holes (FTMHs) are a prominent cause of vision reduction in MacTel type 2, and the standard care for an FTMH is pars plana vitrectomy (PPV) to restore the FTMH and best-corrected visual acuity (BCVA). Olson et al^[5]^ were the first to report the association of an FTMH in the eyes of patients with MacTel type 2 who did not have a surgical intervention. MacTel type 2 is not associated frequently with an FTMH, and thus, reports evaluating the outcomes of PPV for its treatment were few.^[6–11]^ The surgical outcomes in those reports were not good, with a lack of closure or a reopening of the FTMH, compared to those with an idiopathic FTMH.^[6–11]^

Michalewska et al^ [12]^ reported on the effectiveness of the inverted internal limiting membrane (ILM) flap technique to treat refractory idiopathic FTMHs, and this method prevented the reopening of the MH. Sborgia et al^[13]^ were the first to report on the effect of the inverted ILM-flap technique in treating an FTMH associated with MacTel type 2. They reported that this technique resulted in a successful closure of the FTMH, with an improvement of vision to 20/20.^[13]^ However, their report had only 1 patient, with a short follow-up period of 3 months. The outcomes of this technique to treat an FTMH associated with MacTel after a long postoperative period have not been determined.

Thus, this study aimed to determine the surgical outcomes of PPV with the inverted ILM flap technique for the treatment of FTMHs with a 2-year postoperative follow-up in three patients with MacTel type 2.

## 2 Case presentation

The procedures conformed to the tenets of the Declaration of Helsinki, and a signed informed consent was obtained from all subjects after a full explanation of the procedures to be used and the possible complications.

### 2.1 Patient A

**Figure 1:**
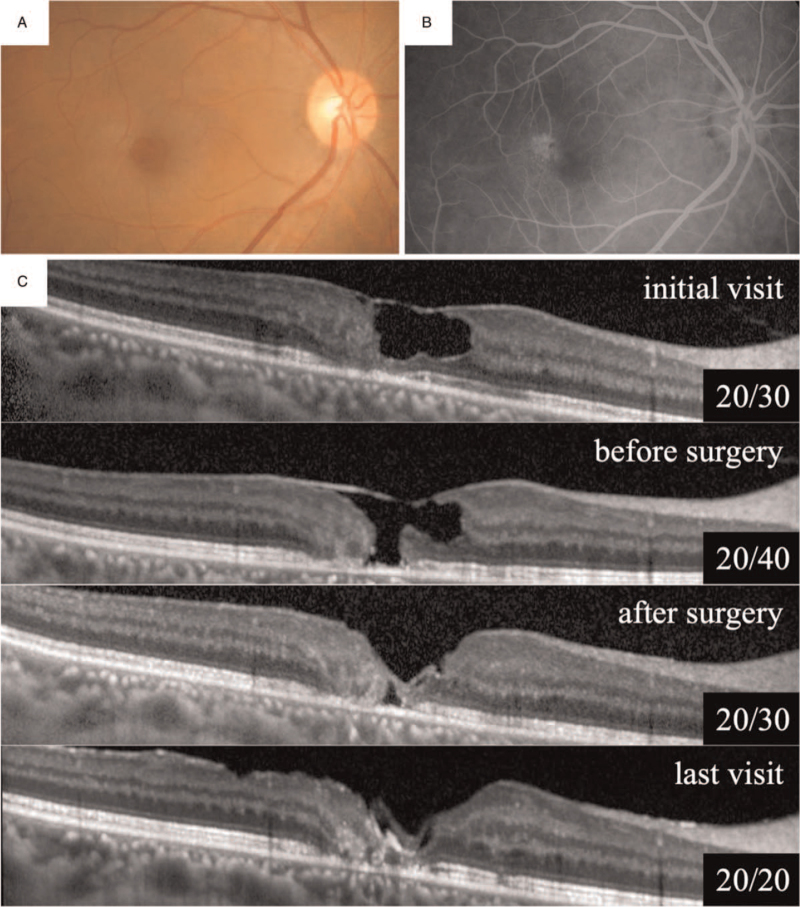
Patient A. Fundus photographs (A) and fluorescein angiograms (FA; B) show venules running at right angles, telangiectatic vessels, and juxtafoveal hyperfluorescence in the right eye. Optical coherence tomography (OCT) images show a large cavitation in the macula at the initial visit (C). Later, preoperative OCT of the right eye shows a full-thickness macular hole (FTMH). After pars plana vitrectomy (PPV), the FTMH is closed and remained closed for 2 years after the surgery.

This is the case of a 58-year-old woman who had metamorphopsia in the right eye for 5 years. The symptoms worsened, and she was referred to our hospital without any treatment. At the initial examination, her BCVA was 20/30 in the right eye and 20/20 in the left eye. Ophthalmoscopy showed that both eyes, and especially the right eye, had a slight graying and opacification of the temporal juxtafoveal macula, with veins running at the right angle to the horizontal axis. A punctate intraretinal pigmentation was also noted (Fig. 1A). Fluorescein angiography demonstrated temporal capillary telangiectasis in the early phase and intraretinal staining in the later phases (Fig. 1B). Optical coherence tomography (OCT) showed a large retinal cavitation in the foveal area of the right eye, but no abnormal findings were observed in the left eye (Fig. 1C). The patient was diagnosed with MacTel type 2 in both eyes.

Although the vision worsened to 20/40 in the right eye 1 year after the initial visit, OCT demonstrated an FTMH with tethered edges, without significant subretinal fluid or retinal edema. There was an absence of the outer retinal layers centrally with an unroofing of the extremely thinned inner retina with no posterior vitreous detachment and without significant traction. After obtaining written informed consent, the patient underwent 25-gauge PPV in the right eye. After the core vitrectomy, the ILM was stained with brilliant blue G (BBG) and peeled off. Then, the ILM flap was inverted and placed over the MH. Then, fluid–gas exchange with 20% SF_6_ was performed to tamponade the retina, and the patient was instructed to maintain a prone position for 1 week after the uneventful surgery. Postoperatively, the OCT findings showed that a small retinal cavitation remained on the fovea. OCT also showed a closed FTMH in the right fovea, which was maintained during the 2-year follow-up period. The BCVA improved to 20/20.

### 2.2 Patient B

**Figure 2:**
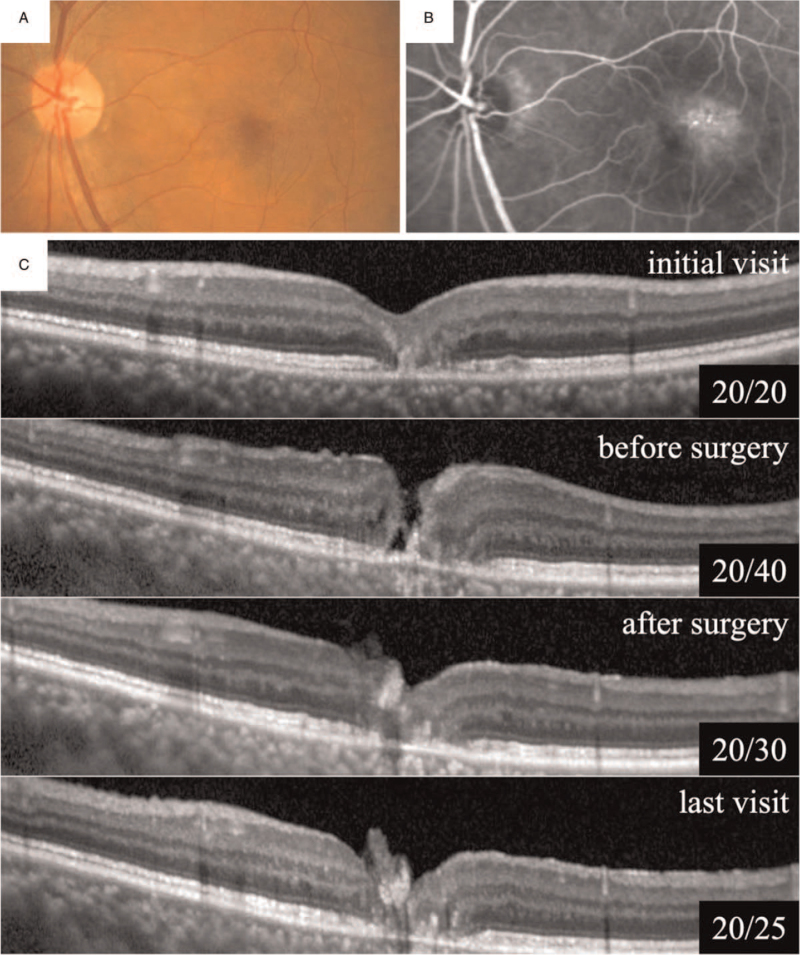
Patient B. Fundus photographs (A) and FA (B) show right-angled venules, telangiectatic vessels, and juxtafoveal hyperfluorescence in the left eye. OCT shows disruption of outer retinal layer in the fovea at the initial visit (C). Later, preoperative OCT of the right eye shows FTMH. The FTMH is closed postoperatively and hyperreflective striae of the ILM can be seen overlying the hole. The FTMH remained closure 2 years after surgery.

A 76-year-old man had blurred vision in his right eye for several years. He had not been treated and was referred to our hospital. At the initial examination, his visual acuity was 20/100 in the right eye and 20/20 in the left eye. Dilated ophthalmoscopy revealed a slight graying and opacification of the temporal juxtafoveal macula with telangiectatic parafoveal vessels and right-angle venules in both eyes (Fig. 2A). Fluorescein angiography demonstrated leakage from a choroidal neovascularization (CNV) in the right eye and slight intraretinal staining in the left eye (Fig. 2B). OCT showed a large retinal cavitation on the foveal area of the right eye and a disruption of the outer retinal layer of the left eye (Fig. 2C). The patient was diagnosed with MacTel type 2 in both eyes. Intravitreal anti-VEGF injection and PDT treatment were performed to treat the CNV in the right eye.

An FTMH was developed 7 years after the initial visit, and the BCVA was reduced to 20/40 in the left eye. OCT showed a thin ERM at the left fovea without significant traction. After obtaining written informed consent, the patient underwent 25-gauge PPV with the inverted ILM-flap technique in the left eye. The patient was instructed to maintain a prone position for 1 week after the uneventful surgery. OCT findings showed that the FTMH was closed postoperatively and hyperreflective striae consisting of the ILM could be seen overlying the hole in the left eye. No remarkable changes were observed in the left fovea during the 2-year follow-up period, and the vision improved to 20/25.

### 2.3 Patient C

**Figure 3:**
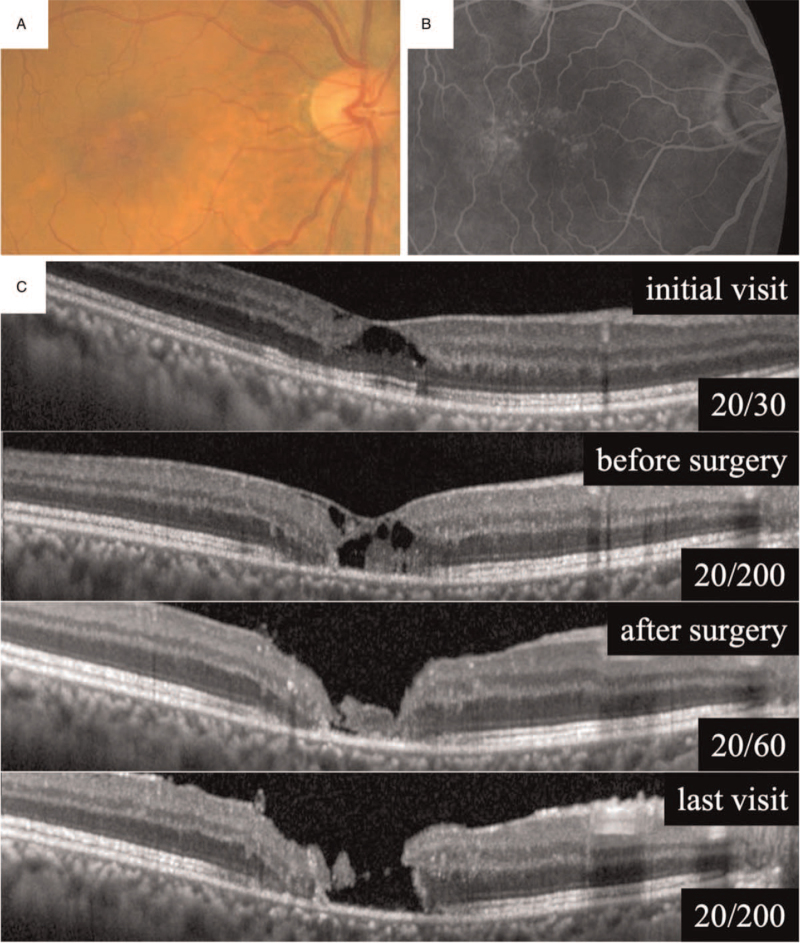
Patient C. Fundus photographs (A) and FA (B) show right-angled venules, telangiectatic vessels, and juxtafoveal hyperfluorescence in the right eye. OCT shows a cavitation at the initial visit (C). Later, preoperative OCT of the right eye shows FTMH. Postoperatively, the hole was closed while small retinal cavitation remained on the fovea. Retinal atrophy progressed and MH was reopened 6 months after surgery.

A 62-year-old man had metamorphopsia in both eyes for 6 months, and he was referred to our hospital without any treatment. At the initial examination, his BCVA was 20/30 in the right eye and 20/15 in the left eye. Dilated ophthalmoscopy revealed telangiectatic parafoveal vessels and venules at right angle in both eyes (Fig. 3A). Fluorescein angiography demonstrated temporal capillary telangiectasis in both eyes and slight intraretinal staining in the late phase (Fig. 3B). OCT showed a retinal cavitation at the fovea in both eyes (Fig. 3C). The patient was diagnosed with MacTel type 2 in both eyes.

He was not treated, and a FTMH was developed 1 year after the initial visit, and his BCVA decreased to 20/200 in the right eye. After obtaining written informed consent, the patient underwent 25-gage PPV with the inverted ILM-flap technique with 20% SF6 were performed to repair the macular hole. Postoperatively, the macular hole was closed while a small retinal cavitation remained at the fovea and vision improved to 20/60. Although the retinal cavitation became smaller with time, the retinal atrophy progressed and the MH was reopened 6 months after the surgery. The vision was decreased to 20/100 in the right eye.

## 3 Discussion

An FTMH is not a frequent complication of MacTel type 2. Olson et al^[5]^ were the first to report the presence of FTMHs in the eyes with MacTel type 2 that had not been treated. Only a few reports exist addressing the outcomes of surgical management for the eyes with MacTel type 2.^[6–11]^ PPV with ILM peeing is a well-established surgical technique to treat an idiopathic FTMH, and it has a 90% success rate. Conversely, the anatomical outcome for the treatment of FTMHs associated with MacTel type 2 is poor. Karth et al^[10]^ reported that their patients with previous reports on surgical interventions^[6–9]^ had an initial anatomical closure rate of 50%, with 30% remaining closed at the last follow-up examination and 20% of the holes initially closed but eventually reopened.^[10]^ Recently, Miller et al^[11]^ reported that 4 of 12 eyes had a closure of the FTMH associated with MacTel type 2.

The inverted ILM-flap technique reduces the chance of an open MH in the eyes with refractory idiopathic FTMH.^[12]^ Sborgia et al^[13]^ reported a single case of MacTel type 2 with an FTMH that had been treated by PPV using the inverted ILM-flap technique. They reported a successful closure of the FTMH and an improvement of vision to 20/20.^[13]^ However, the follow-up period was relatively short at 3 months. Thus, the outcome after a long follow-up period has still not been determined.

The inverted ILM-flap technique was used to treat an FTMH in 3 cases of MacTel type 2, and the outcomes were followed for at least 2 years after the surgery. The FTMH was closed successfully in all of three patients after the surgery, but the FTMH in one patient reopened at 6 months. The hole in the other 2 patients remained closed for at least 2 years after the surgery. Thus, our rate of closure of the FTMH in our 3 cases suggests that this technique is as good as that of earlier studies,^[6–11]^ although the number of patients was low.

The precise pathophysiology of the formation of an FTMH in the eyes with MacTel is still not determined. Degeneration and atrophy of the Müller cells may be related to the development of an FTMH.^[14,15]^ The ILM may play a role as a scaffold for the proliferating tissues and the migration of activated Müller cells that promote the closure of a macular hole by producing neurotrophic factors.^[16]^ The results of Sborgia et al^[13]^ and our study suggest that the inverted ILM-flap technique would be an effective treatment not only for idiopathic FTMH but also for FTMH associated with MacTel. Thus, the inverted ILM-flap technique is effective for the closure of a FTMH associated with MacTel type 2. However, patient 3 had a reopening of the FTMH because of retinal atrophy at the fovea. These results indicate that the surgery is helpful for the closure of an FTMH with MacTel type 2, but it cannot prevent a reopening of the hole when the retinal atrophy progresses.

Our results showed that the vision was improved to 20/20 and 20/25 in the 2 patients in whom the FTMH remained closed. No significant change in the BCVA between the PPV treatment group and medically managed eyes has been reported previously, but the FTMH was closed in only four eyes of 12 eyes in that study.^[11]^ On the other hand, Sborgia et al^[13]^ reported that the vision was improved to 20/20 in the eye with closure of the macular hole by the inverted ILM-flap technique. In the eyes with an idiopathic FTMH, the closure of the FTMH can lead to a significant improvement of vision. Vision improvement in the eyes with FTMHs associated with MacTel may also be related to the anatomical closure. However, additional investigations are needed to determine the correlation between the anatomical success and the BCVA on a larger number of patients with FTMHs associated with MacTel.

This study has several limitations. An important limitation was that we studied only three patients with MacTel type 2. However, this retinal disorder is rare and not many cases are examined. In addition, cataract surgery combined with PPV was performed. Although the grade of cataract was mild in all of the cases, the extraction of the cataracts may have influenced the improvement of the BCVA. Second, only the vision was measured to assess the function of the fovea before and after the surgery. The BCVA does not represent the changes of all the visual function of the fovea. Moreover, paracentral scotoma can progress in the eyes with MacTel type 2, and evaluating the function with another measurement, for example, microperimetry or visual fields, is better.

## 4 Conclusion

The inverted ILM-flap technique may be an effective and safe treatment for an FTMH in patients with MacTel type 2. However, additional studies on patients using the ILM inverted flap technique are needed to confirm our findings.

## Author contributions

**Conceptualization:** Shungo Nishiyama, Takeshi Iwase.

**Data curation:** Shungo Nishiyama.

**Funding acquisition:** Takeshi Iwase.

**Methodology:** Shungo Nishiyama, Takeshi Iwase.

**Supervision:** Takeshi Iwase.

**Writing – review & editing:** Shungo Nishiyama, Takeshi Iwase.

**Writing – original draft:** Takeshi Iwase.

**Conceptualization:** Shungo Nishiyama, Takeshi Iwase.

**Data curation:** Shungo Nishiyama.

**Methodology:** Takeshi Iwase.

**Supervision:** Takeshi Iwase.

**Validation:** Takeshi Iwase.

**Visualization:** Takeshi Iwase.

**Writing – original draft:** Shungo Nishiyama, Takeshi Iwase.

**Writing – review & editing:** Shungo Nishiyama, Takeshi Iwase.
